# Regional engagement and spatial modelling for natural resource management planning

**DOI:** 10.1007/s11625-015-0341-5

**Published:** 2015-10-08

**Authors:** Wayne S. Meyer, Brett A. Bryan, David M. Summers, Greg Lyle, Sam Wells, Josie McLean, Mark Siebentritt

**Affiliations:** 1 Environment Institute, University of Adelaide, Adelaide, SA 5005 Australia; 2 Landscape Systems, Biological Sciences, University of Adelaide, PMB 1, Glen Osmond, SA 5064 Australia; 3 CSIRO, Waite Campus, Urrbrae, SA 5064 Australia; 4 The Australian National University, Canberra, ACT 0200 Australia; 5 University of Adelaide, Adelaide, SA 5005 Australia; 6 Seed Consulting Services, 106 Gilles Street, Adelaide, 5000 Australia

**Keywords:** Transdisciplinary, Envisioning, Values-rich narrative, Landscape futures analysis, Management options, Regional planning

## Abstract

Changing unsustainable natural resource use in agricultural landscapes is a complex social–ecological challenge that cannot be addressed through traditional reductionist science. More holistic and inclusive (or transdisciplinary) processes are needed. This paper describes a transdisciplinary project for natural resource management planning in two regions (Eyre Peninsula and South Australian Murray-Darling Basin) of southern Australia. With regional staff, we reviewed previous planning to gain an understanding of the processes used and to identify possible improvement in plan development and its operation. We then used an envisioning process to develop a value-rich narrative of regional aspirations to assist stakeholder engagement and inform the development of a land use management option assessment tool called the landscape futures analysis tool (LFAT). Finally, we undertook an assessment of the effectiveness of the process through semi-structured stakeholder interviews. The planning process review highlighted the opinion that the regional plans were not well informed by available science, that they lacked flexibility, and were only intermittently used after publication. The envisioning process identified shared values—generally described as a trust, language that is easily understood, wise use of resources, collaboration and inclusiveness. LFAT was designed to bring the best available science together in a form that would have use in planning, during community consultation and in assessing regional management operations. The LFAT provided spatially detailed but simple models of agricultural yields and incomes, plant biodiversity, weed distribution, and carbon sequestration associated with future combinations of climate, commodity and carbon prices, and costs of production. Stakeholders were impressed by the presentation and demonstration results of the software. While there was anecdotal evidence that the project provided learning opportunities and increased understanding of potential land use change associated with management options under global change, the direct evidence of influence in the updated regional plan was limited. This project had elements required for success in transdisciplinary research, but penetration seems limited. Contributing factors appear to be a complexity of climate effects with economic uncertainty, lack of having the project embedded in the plan revision process, limited continuity and capacity of end users and limited after project support and promotion. Strategies are required to minimise the controlling influence that these limitations can have.

## Introduction

There is increasing awareness that human use of, and effect on, the Earth’s natural resources is either operating beyond or trending to exceed the boundaries that are deemed sustainable for the foreseeable future (Rockstrom et al. 2009; Steffen and Stafford Smith 2013). Accompanying this increased awareness is the realisation that addressing these global social–ecological issues is complex (Holling 2001) and will not be addressed with reductionist science methods (Dedeurwaerdere 2014). Hence, there is increasing interest in multi- and trans-disciplinary applications that take a holistic and systems-wide perspective (Lang et al. 2012; Mauser et al. 2013; Rice 2013). The breadth of systems perspectives can be from global to local. However, for changes in resource use to be effective, they will need to be implemented at the level of decision-making responsibility. Polycentric governance principles devolve decision making to the lowest level that can effectively discharge it (Lane et al. 2009; Bryan et al. 2013). In Australia, management of natural resources (soil, water, biota) has been devolved to the regional level following trends towards integrated catchment management (ICM) of river basins similar to those operating in Europe (Boonstra and van den Brink 2007; Bocher 2008) and watershed management in North America (Michaels 2001; Margerum and Whitall 2004). Regional agencies have been variably vested by State and Australian governments with the responsibility of planning and implementing programs to repair, maintain and protect the soil, water, and biological resources. Here, we describe a process that was designed to enhance regional natural resource management agency engagement with complex scientific information, and help planning and operations have greater and more enduring effectiveness.

Regional planning needs to be scientifically informed because the inter-dependency of natural resource elements and effects of their use are recognised as complex (Harris 2007; Norberg and Cumming 2008). Complexity becomes more acute when the interactions with the social settings of economics, community preferences, governance, and policy are considered and operating within the context of global and regional change (Burgi et al. 2004; Sheppard et al. 2011; Bryan et al. 2013). Projections of the future generally involve complex scientific and geographic information, and are highly uncertain which can lead to stakeholder intimidation (Carmichael et al. 2004), scepticism and, ultimately, rejection of proposed plans. Hence, the investigation, planning and implementation processes that should become part of adapting to changed conditions need to be multi- and trans-disciplinary in character (Robinson et al. 2006; Roux et al. 2010; Lang et al. 2012; Mauser et al. 2013; Rice 2013; Campbell et al. 2014; Dedeurwaerdere 2014). The lack of appreciation of the interacting effects of social, environmental and economic influences is likely to be a major reason why improved condition of regional resources in Australia is hard to identify even after a decade or more of directed activity (Williams et al. 2008). Reviews of NRM planning and operations found that even though science-based evidence and projections were available to many regions, much of this was not used or only cursorily used (Chartres et al. 2004; Williams et al. 2008). There are many other reasons that contribute to the apparent lack of general improvement (Curtis et al. 2014). Among them is the small amount of money for works relative to the spread and magnitude of resource degradation, and the limited social and human capital available to lead and effect change. The context can be generalised as a social ecological setting, grappling with complex environmental, economic and community issues most often with limited financial and human resources.

Several approaches to increasing the influence of environmental science to landscape planning and management have been described including multi-criteria analysis (Bryan 2010), deliberative evaluation (Bryan and Kandulu 2011), expert panel consultation/involvement, co-design ideas and companion modelling (Summers et al. 2015). In addition to these technically oriented interactions, there are other areas of social research directly relevant to regional planning and natural resource management, such as analysis of social and institutional structures, stakeholder agency and power, and the use of participatory methods such as scenario planning and adaptive governance (Voß and Bornemann 2011; Plieninger et al. 2013; Reed et al. 2013; Wyborn 2015). Other works in this field have focussed on the importance of identifying the underlying core values of stakeholders. For example, Raymond et al. (2009) and Hatton MacDonald et al. (2013) described an interview methodology to identify the values of community leaders in the South Australian Murray-Darling Basin NRM region that they would apply to decisions about “multiple-use landscapes” in their region. As explored by Lejano et al. (2013), the values that people hold particularly in relation to the environment are often powerfully expressed through narratives that illustrate their connection with their surrounds. The narratives that accompany different community influenced future scenarios will almost certainly reflect commonly held values although these may not be explicit. The reason for the focus on values stems from the assertion that without a better understanding of the values of the stakeholders “public policy … may consistently fall short of expectations” (Hatton MacDonald et al. 2013). Lejano et al. (2013) also asserted “that stories shape how we behave, and that in paying attention to our stories we can better understand—and change—our behaviour”. The fundamental importance of identifying stakeholder values as the major influence in the likely success of change (in behaviour) in complex social-ecological systems was explored by Wells and McLean (2013). For operating in “the paradigm of complexity”, they set out a methodology (One-Way Forward) that has four central components—“envisioning, core messages (values), indicators of progress, and experimentation”. However, there are few examples of their direct influence on planning and even fewer that attempt to measure success or failure.

In this study, we combined the One-Way Forward envisioning process together with a web-based tool to connect regional planners with complex scientific information for enhancing their planning processes under global change. We implemented this with two regional natural resource management agencies in South Australia. A multi-disciplinary researcher and stakeholder representative steering group identified a four-stage project to develop an improved science-informed, information-rich and enduring process for regional natural resource management planning. First, with regional NRM people we reviewed past planning processes and outputs to understand the elements that they thought worked well and those that had not. Envisioning was then conducted to engage regional stakeholders, to connect them at the level of shared values, and to identify the key design components of a decision support planning tool for communicating complex planning information. We then built and delivered a web-based tool that presented simplified science-based management options along with the likely consequences and trade-offs and enabled stakeholders to explore and engage with this information. Finally, we evaluated the success of the process during training sessions and then using a series of semi-structured stakeholder interviews. In this paper, we describe the process, evaluate its success, and discuss the implications for better engagement between science and management.

## Project development and methods

**Fig. 1:**
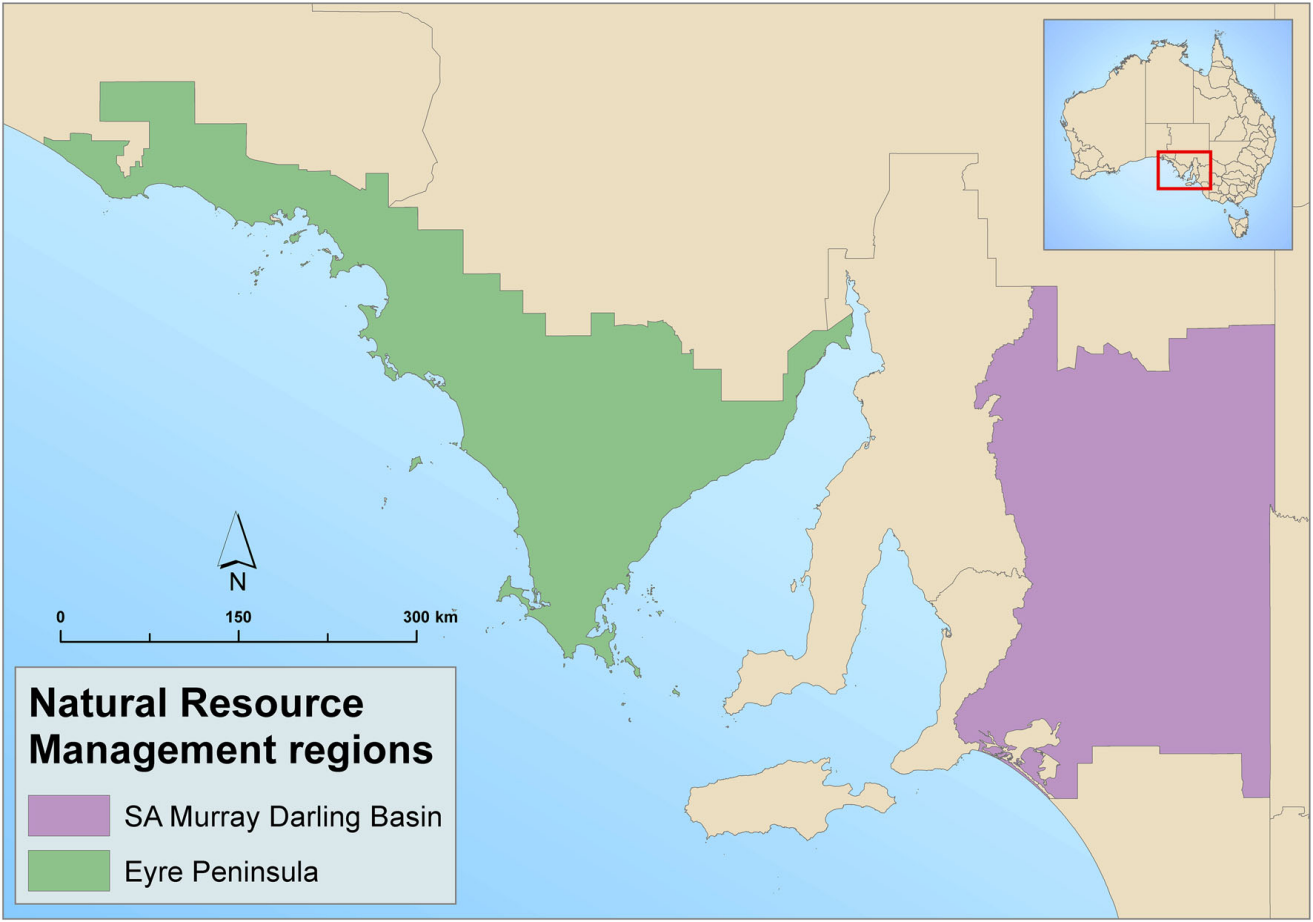
Location of the project areas

The project focussed on two community-based, regional NRM agencies in South Australia (Fig. 1)—the South Australian Murray-Darling Basin Natural Resource Management (SAMBD) region and the Eyre Peninsula Natural Resource Management (EP) region. Regions are governed by a Board appointed by the State Government, which directs the regional management staff. Both regions are predominately semi-arid and sparsely populated. They are primarily agricultural with rain-dependant grain and pasture for sheep grazing. Prior to European settlement after 1840, the native vegetation cover was open Eucalypt woodland. Much of this woodland has been cleared for agriculture between the end of WWII and the instigation of clearance regulations in the early 1980s. In the SAMDB, irrigated horticulture and viticulture occur in a narrow ribbon development along the River Murray, while in the EP region ocean fisheries and aquaculture, and land-based mining are increasingly important economic activities.

This research coincided with the time that NRM regions were required to revise their existing strategic plan and develop their next 5-year plan. The SAMDB’s first plan had been partially informed by information generated from a large, integrated, spatially explicit assessment of regional futures called the Lower Murray Landscape Futures (Bryan et al. 2007). Subsequently, the modelling of future land use scenarios was refined and described in general terms as Landscape Futures Analysis (LFA) (Bryan et al. 2011) with the analysis being applied to other NRM regions (Bryan and Crossman 2008; Pettit et al. 2011).

For this project, a deliberative and adaptive staged process was designed to respond to stakeholder needs and directions. Hence, some process and technical input were modified as the project progressed, i.e. the results from an earlier stage informed the methods in a subsequent stage. A summary is given in Table 1. A project steering group provided input to, and oversight of the research, and assisted in connecting the research to other NRM activities and stakeholders. The group included six researchers from different disciplines, a senior manager and the planning manager from the two NRM regions, and two independent advisors and facilitators.

**Table 1:** Summary of the project process from inception to completion of the landscape futures analysis tool

Stage	Activity involvement and output
1. Review previous planning process	
	Identify people involved in the first strategic plan to form a focus group
	Facilitated meetings to review first planning process and identify improvements
	EP Region: 7 people
	SA MDB Region: 7 people
2. Define regional aspirations	
	Facilitated context information and envisioning meetings to develop a shared vision narrative addressing “how do you want to experience the regional landscape”
	Adelaide: 27 people
	SA MDB Region: 38 people
	EP Region: 32 people
	Follow-up meeting in each region to develop indicators of progress—involved core groups of 5–7 people nominated by the regional manager and the steering group. Four people from SA MDB region and 3 from the EP region in the core group had been involved in the review and envisioning meetings
	Identify requirements of the biophysical descriptions of the region that are needed to be analysed to provide possible land use options, consistent with aspirations, for adapting to future change
3. Collate data and analyse bio-physical data to develop the landscape futures analysis tool	
	Regional data for climate (20 + stations, 50 years), soil descriptions and distributions, land use, cadastral information
	Regional data of agricultural production (yield, annual cost and return statistics)
	Herbarium records of endemic and weed plant species distribution and abundance
	Recent records of dry matter yields from tree plantings for carbon sequestration
	From modelling outputs display values and spatial distribution of yield and financial returns from agricultural production and carbon sequestering tree plantations in response to climate, cost and return scenarios
	Display model outputs of endemic and weed plant species distributions in response to climate scenarios
	Two meetings in each region to demonstrate the prototype LFAT and subsequently adjust the user interface and form of output
4. Provide training and assess effectiveness of process	
	Project content and outputs were presented to the Board and relevant staff in both regions
	Tutorial sessions on the use of LFAT were given to staff in both regions
	Follow-up activity using the LFAT was commissioned by EP NRM
	Anecdotal evidence from regional NRM staff of project influence collated
	Review of first and revised strategic plan documents for evidence of influence

### Stage one: review of previous planning process

We undertook a context review of the planning process used to develop the first regional strategic plan. The deputy general manager of the EP Board (2005–2009) and the planning and evaluation manager of the MDB Board (2005–2013) identified those individuals who had the knowledge and experience to critically review at least one of the following four areas relevant to regional plan development:

- Processes used to develop the first regional plan, with a specific emphasis on the community consultation process, the structure of the plan and timeline of plan development;
- Information and data sourced to inform the development of the plan;
- Scientific tools used to inform the development of the plan, as well as future directions in tool development;
- Evaluation of the effectiveness of the plan in driving NRM change and the level of ownership of the plan in the region.

The project team in collaboration with senior managers in each Board developed a series of questions to guide the focus group discussion. An indication of the decision levels that meeting and workshop participants held is given in Table 2.

**Table 2:** Stakeholder analysis of meeting attendees

Meeting description	Decision level	Number of people
Review of first planning process		
Eyre Peninsula NRM Region		
NRM Board Member	P	2
Planning and Evaluation Manager	E, M	1
Planning assistant	O	2
GIS and operational technician	O	2
SA MDB NRM Region		
Deputy CEO	P, E	1
Community Advisory Group member	P	1
Planning and Evaluation Manager	E, M	1
Planning assistant	O	2
GIS and operational technician	O	2
Envisioning Workshops		
Adelaide		
Australian Government NRM Agency	P	2
Australian Government Research Agency	E	1
SA Government Department of Environment	P, E	4
SA Government NRM Council	P	2
NRM Board members	P	5
NRM Board Staff	E, O	10
Community Advisory Group Members	C	3
SA MDB NRM Region		
SA Government Department of Environment	P, E	2
NRM Board members	P	3
NRM Board Staff	E, O	8
Community Advisory Group Members	C	15
Local Government	E, O, C	4
Grower and Community Organisations	C	6
EP NRM Region		
SA Government Department of Environment	P, E	1
NRM Board Members	P	3
NRM Board Staff	E, O	9
Community Advisory Group Members	C	4
Local Government	E, O, C	3
Grower and Community Organisations	C	12
Indicators of Progress		
Eyre Peninsula NRM Region		
NRM Board Member	P	1
Planning and Evaluation Manager	E, M	1
Community Advisory Group Member	P	1
Planning Assistant	O	2
GIS and operational technician	O	2
SA MDB NRM Region		
Deputy CEO	P, E	1
Community Advisory Group Member	P	1
Planning and Evaluation Manager	E, M	1
Planning Assistant	O	2
GIS and Operational Technician	O	2
LFAT Prototype and Training (×2)		
Eyre Peninsula NRM Region		
Planning and Evaluation Manager	E, M	1
Planning Assistant	O	2
GIS and Operational Technician	O	3
SA MDB NRM Region		
Deputy CEO	P, E	1
Planning and Evaluation Manager	E, M	1
Planning Assistant	O	2
GIS and Operational Technician	O	4

Decision levels: *P* policy, *E* executive, *M* managerial, *O* operational, *C* community

### Stage two: envisioning to identify values and guide information need and form

Three envisioning workshops were directed at engaging with multiple decision-making levels (Table 2) to identify their aspirations with respect to the expectation of the engagement process and how they wanted to experience the landscape. The workshops involved as many people as possible who were associated with the regional planning process. The intention was to facilitate interaction between a hierarchy of influencers from Australian and State Government agencies through to the regional NRM Boards and planning staff. A brief context setting session highlighted the global, national (Bryan et al. 2013) and regional economic and biophysical influences that were thought to affect the region. Then, following the method of Wells and McLean (2013) a random set of photographic images was used to facilitate the development of a narrative from the participants that described what those involved really wanted from the region. After the initial workshop, it was apparent that a greater level of explanation was needed to help people gain a better understanding of the link between the envisioning and engagement process and the ongoing planning process. Many of those involved were aware of the complexity of considerations in regional planning. This complexity was increased by identifying that future climate and commodity price scenarios should be part of the planning. A brief explanation was developed of the project intentions and the role of envisioning in helping provide direction that the plan should take. The explanation was subsequently distributed to workshops that followed in the two regions. The central question being explored was how to work with people in a way that connected visions of their desired futures (and the values embedded within those visions) with decisions informed by the science, so that a narrative describing the attributes of a more sustainable future planning process emerged.

### Stage three: define and refine regional data for planning option assessment tool

This stage focussed on the development of a regional information capture and projection “tool” (subsequently called the Landscape Futures Analysis Tool, LFAT). A preliminary design specification for LFAT came from the discussions about previous planning and the envisioning process. The main concerns associated with the previous planning process were the need for better science information and more interactive and transparent management option assessment processes. The envisioning process identified the need for openness, inclusiveness and involvement. Additional consultation with regional planners settled on four key NRM planning issues, viz:

- Conserving biodiversity—managing remnant native vegetation and restoring corridors;
- Managing weeds—with targeted monitoring of future invasion risk hotspots;
- Storing carbon—finding the best places for carbon sequestering plantations as part of exploring opportunity for vegetation and financial diversity;
- Agricultural production—quantification of yields and its distribution in the region and over time.

### Stage four: demonstration and assessment of process effectiveness

This stage had two related elements. The first element included a beta test of the web-based LFAT through demonstration and feedback at two workshops in each of the regions involving 3–8 operational staff. Subsequently, a tutorial demonstrating the essential features and functions of the LFAT was prepared to assist and encourage end user assessment. Opportunity for interaction existed post-release of the tool through online and personal contact. The second element was the informal and ongoing assessment of the process and the usefulness of the tool. Semi-structured interviews were conducted with the planning managers in each region, with the former regional managers, and with a former Board member in one region and an operations manager in the other region, all of whom had involvement with the project. Anecdotal observations were noted during ongoing interactions with Board staff. A comparison of the content of the first regional natural resource management plan and that of the revised plan was made to assess the influence or otherwise of the project.

## Reviewing the planning process

The initial engagement to identify what had been done during the previous planning process was welcomed and enthusiastically participated in. There was a sense that some of those involved were pleased to identify constraints and opportunities that could benefit future planning processes. The following observations were drawn:

- the main concerns related to perceived weaknesses of integration, accountability and capability in the planning process;
- different levels of capability were evident between the regions and both plans were adjudged as “not being well informed by the best available science”;
- both regions were concerned at the lengthy time taken to develop the NRM plans, and their relevance;
- both regions identified that the plans were inadequate at providing direction when opportunistic funding from Australian and State Government agencies became available;
- both regions questioned the merit of the written regional NRM plan—few people read it, few used it to guide decisions, there was little local community ownership of it and the evidence was that the plan did not primarily drive the NRM Board’s business—hence the worth of nearly 4 years of financial and intellectual investment to develop the plan was questioned;
- as part of the scepticism on the value of the NRM plan, there was a sense of general apathy towards the plan development process by those involved in it—i.e. “people did not seem to care post-plan development”.

## Envisioning to identify values and guide information need and form

The important element of this approach was that it sought to identify the values that people inevitably use in making decisions but which are rarely made explicit. The primary values expressed by workshop participants centred on trust, openness, inclusiveness, clarity and enjoyable learning.

With the input from the envisioning workshops in the two regions, a narrative that described the values was developed and circulated to the participants. At the follow-up meetings, these values were highlighted and descriptions of indicators of progress were developed. These were couched in terms of “what would you observe if your vision was being lived now?” that would provide evidence that the important values associated with the planning process were being acknowledged. A summary of the values and associated indicators of progress is given in Table 3.

**Table 3:** Example of narrative points for how people wanted to experience the planning process for their regional landscape. These include explicit, generally held values. Indicators of progress associated with the “values” are included

Process and experiencing the landscape “values”	Indicators of progress
1. Trust—must exist and is central	
	Those that come are willing to participate and want to stay in the conversation in whatever way they want
	We will observe diverse contributors
	We will see people seeking to understand by listening and asking questions
	We will observe that everyone feels they have the opportunity to participate
	We will observe an openness to ‘opposed’ and new uses without ‘battlelines’
2. Language—use a common language that everyone can understand	
	People take care with language and explain (and check for understanding) technical terms if and only if they must use them
	Non-technical people participate in the conversation demonstrating that everyone has understood clearly
	Content is tailored to anticipate the audience response—audience feels that content is relevant (local language, local stories)
3. Wise use—of natural resources	
	We will hear conversations about ‘wise use’ of natural resources
	People informing their decisions and actions with all relevant knowledge (including Landscape Futures Analysis)
4. Interlinking is critical—collaboration recognises interlinking of interests and relationships	
	Different voices/perspectives are reflected in the plan that recognise mutual interests and opportunities (valuing diversity and alert to synergy)
	We will observe collaboration of ‘strange bedfellows’
5. Inclusive—of scientific and traditional knowledge, complexity, diversity will create a safe environment for robust discussions	
	Willingness to air and explore ‘knowledge’ from diverse sources (e.g. local, scientific, traditional) and everyone comes away with a sense of learning something new
	General endorsement of the planning process by participants—and of planning proposals by regional decision makers

While the envisioning process was primarily designed to improve engagement with decision makers and end users (stakeholders), it sought, through a heightened sense of involvement and ownership by stakeholders, to gain a good understanding of the form and type of information that would be most helpful in regional NRM planning. This critical aspect of the envisioning process encouraged the participants to describe their important values with tangible examples from aspects of regional life and local resources. These highlighted the importance of sustained and diverse regional production to maintain a viable community and the importance of looking after the soil, water and biodiversity assets as people valued these as part of their sense of place. The uncertainty of climate conditions was often used to emphasise the importance of local and traditional knowledge in adapting to variable conditions. Threats to the sense of regional continuity were often couched in descriptions of uncertainty in terms of trade (prices received relative to costs), uncertainty of future climate and being overwhelmed by invasive species if technological solutions were not forthcoming.

From these discussions and from the review of the initial planning process, the project team refined the proposal for an information tool. Features which were agreed to be important included:

- a focus on the condition of regional natural resources at a scale which was relevant to local communities,
- explicit representation of future uncertainty in both climate and terms of trade,
- flexibility to incorporate existing knowledge and be updated,
- use of terms and with outputs that are understood by stakeholders,
- use of best available science to inform the processes included in future projections,
- transparency and communication about the development process and checking the credibility of outputs with regional stakeholders.

Regional NRM staff involved in the project was enthusiastic about the prospect of an analysis tool that was regionally specific, was climate, soil and vegetation informed, could be used to develop scenarios and hence inform plans, could have outputs for visually demonstrating planning options to stakeholders and could be regularly updated. These attributes coincide with the values expressed at the envisioning workshops of openness, with a capability to adapt and adjust and provide a way of dealing with complexity and uncertainty associated with climate and terms of trade.

## Defining and refining regional data for planning option assessment tool

The tool is described in detail by Summers et al. (2015). Briefly, LFAT was built around agricultural production, carbon sequestration, biodiversity distribution, and weed distribution. Each of these modules used outputs from simple models of yields (of grain and carbon) and of occurrence and abundance of plant species. The agricultural production module used a system modelling approach (Bryan et al. 2011) while conserving biodiversity used species distribution modelling (Crossman et al. 2012; Summers et al. 2012) and an economic cost-benefit approach to inform policy such as targeted incentive schemes under climate change (Crossman et al. 2011; Paterson and Bryan 2012). Managing weeds used species distribution modelling (Bryan et al. 2011) and a risk analysis framework to identify areas at high risk of both agricultural and ecological weed invasions under climate change for targeting monitoring and management efforts. Storing carbon used a landscape planning approach to identify areas that are suitable (and unsuitable) for carbon plantations subject to satisfying several specific criteria. Each of the four issues was implemented as a separate interface in the LFAT. The models were responsive to climate and soil conditions and, hence, projections of possible future climate, price and cost scenarios could be generated. The outputs of user-chosen scenarios were displayed in geographic information system enabled maps. The intention was to have a rapidly responding information tool that could use the extensive regional biophysical and economic data and produce process-informed production and conservation consequences. The objective was to assist stakeholders to assess possible future management options for their region and to have this displayed in an attractive, spatially explicit map form.

**Fig. 2:**
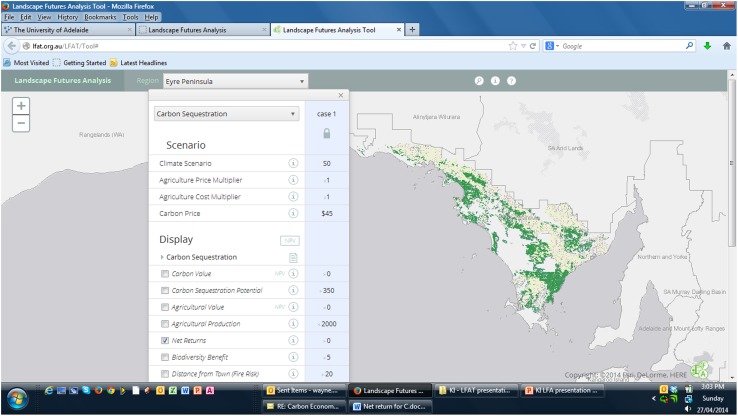
Example screen output from Eyre Peninsula Region displaying the distribution of possible carbon sequestering area with current climate conditions, current returns and costs from agriculture and with a price of carbon at $AU45 per tonne

An example output screen is shown in Fig. 2. The tool (http://www.lfat.org.au/LFAT3) is extendable, as interfaces can be added to address other specific NRM planning issues.

## Demonstrating the planning tool and assessing its effectiveness

A clear intention of this project was to have involvement of people from both regions from inception through to completion. Hence the project steering group had two representatives from each region. Of these two, at least one was involved throughout the project. Meetings within the regions for the first planning process review, envisioning, indicators of progress, tool definition and tool demonstration had one or two people from each region who were involved in all meetings. However, there were changes in management and operational planning staff during the project and hence there was limited consistency in involvement.

Presentations to the regional Boards and to planning and operations staff were well attended and the output of both the process and LFAT was complimented as “impressive”. A summary of the generalisations from the LFA (Table 4) provides an indication of the breadth of information available.

**Table 4:** Summary of generalised output and interpretation from the landscape futures analysis tool

Landscape Futures Analysis illustrates
There are many combinations to consider (>5000)
Regional variation is large and important
Changes in prices and costs have more dramatic effects than climate change (but we can develop a “feel” for the sensitivity of each variable on the land use consequences)
Some locations will have higher productivity as temperatures increase and rain declines
Opportunity for carbon sequestration is location- and price-dependant
Response of plant distribution to climate change is highly species-dependant
Many current reserves are inadequate to conserve native species as climate changes
Using LFA to develop a “climate ready” plan can make it more objective

The tutorial sessions with Board staff nominated as potential users of LFAT were interactive and, on the surface, were well received. However, no further direct involvement with the LFAT site was recorded from the SAMDB region. For the EP, a follow-up contract was completed that took the LFAT grain and carbon yields associated with the various climate, price and cost scenarios and rearranged these within vegetation association subregions rather than an arrangement by similar climatic conditions. This information was added to the LFAT website as a new information layer for the EP Region and used in preparing the new regional plan.

The comparison of the SA MDB NRM Strategic Plan of 2009 (South Australian Murray-Darling Basin Natural Resources Management Board 2009) and for 2014 (Natural Resources SA Murray-Darling Basin 2014) provides some insight into changed thinking over this time. The most recent Plan is strongly couched in terms of managing in a “landscape”, “resilience” and “socio-ecological” context rather than presenting planning and management in a program framework as in 2009. This emphasis on a connected systems approach is followed through with the consideration of the four Local Action Planning Districts described in terms of “Landscapes, Livelihoods and Lifestyles”. These concepts were introduced to the Region during the development of this project. However, the 2014 Strategic Plan makes no reference or acknowledgement of the LFA process. While the possible effects of regional climate change are briefly mentioned in the “atmosphere asset” there is no explicit consideration of climate change on land use or biodiversity. Information on the NRM website (http://www.naturalresources.sa.gov.au/samurraydarlingbasin/projects/climate-change-projects) provides links to “adapting to climate change” projects. One of these projects (Siebentritt et al. 2014) reports on “building resilience to a changing climate” in the region. This project was informed by the LFA process but it does not include any specific examples of outputs from LFAT.

The 2009 Strategic Plan for the EP NRM Board (Government of South Australia 2009) was quite explicit about the connected social–ecological system as expressed through the overarching statement “Natural resources supporting ecological sustainability, vibrant communities and thriving enterprise in a changing climate”. Of the six key objectives required for the new Strategic Plan (http://www.naturalresources.sa.gov.au/eyrepeninsula/about-us/our-regions-plan), two identify the need for simplicity and increased stakeholder ownership, while others identify a sub-regional approach, identification of specific climate change adaptation strategies and “resilience thinking …to assess the interactions and thresholds of economic, social and environmental domains”. The EP NRM Board’s Planning Manager (Sibly, pers. comm. 2015) has indicated that “Our (EP NRM Board) intention is to include some basic outputs from LFAT, and a link to the version 3 so that stakeholders can customize their queries”.

## Evaluating the success of the decision support and planning process

We have presented an adaptive, deliberative process for better engaging regional agencies with science for supporting the planning process. Results from the review of the previous planning process indicated that there was a need for clarity with respect to targeted audience and ongoing function. Part of the issue seems to be the lack of genuine ownership and hence, lack of confidence that the regional NRM plan was well conceived, well informed and adaptable, and truly reflected the aspirations of the stakeholders. This finding was consistent with the impressions that were used to design this research project.

Interaction with many of those involved in setting the need for adaptation planning through to those preparing the revised plans and those charged with implementing plans was very positive. However, making the connection between the process of developing the values-rich narrative from the envisioning workshops and using this to guide subsequent planning and implementation was only partially successful. Some participants felt that the process was too time consuming and did not generate the tangible and specific planning actions similar to those identified during the first regional planning process. It was also evident that some participants were uncomfortable with a focus on identifying values—this was different to the usual methods of engagement that focus almost entirely on biophysical content and only implicitly on personal values, feelings and relationships. Many of these participants became more engaged during explanation and demonstration of the LFAT, i.e. when there were tangible data and projections being discussed. The comments with regard to time and the degree of discomfort may be interpreted as indicators that the participants were engaging in real ‘adaptive change’ and seeking ways to ‘avoid the work’ as can be expected when adaptive work is undertaken (Heifetz and Linsky 2002). Making explicit action links between the envisioning and engagement process and the ongoing planning process may always be problematic for those participants who have little regard for inclusive involvement and prefer to rush to the final plan. Those involved with psychological and human motivation studies assert that it is the discussion and sharing that is the real value (Wells and McLean 2013). While there is little doubt that this has value, the experience of this project suggests that greater direction to participants engaged in the envisioning workshops was needed to encourage examples of local and regional natural resource features, uses and management to illustrate their important values. Additional participative research is needed to improve this aspect.

It was evident to the project team, from the project proposal, inception and development that significant learning and information exchange occurred. This was particularly true in relation to exchange about the role and limitations of modelling possible regional climate and economic futures. From feedback, it was indicated that explanation of the uncertainty associated with the modelling and projections was welcomed and did not indicate “bad science” when there were a range of possible outcomes. However, it was also in this domain that we encountered a sense of consultation “fatigue”—particularly around the uncertainty of projections associated with climate change which troubled many because it signalled the need for change, with possible effects that could not easily be assessed by the stakeholder group.

The lack of guidance from the responsible government agency as to how the regions could direct their management beyond the development of a regional plan is regrettable. Part of this problem comes because the role of the Board in assessing the effectiveness of the existing plan and hence identify improvements in subsequent plans is not clear and is variably considered by different regional Boards (Hopton 2015, Pers. Comm). At an operational level, if the plan and the tools used in its development are not adaptive and flexible enough to account for the changing circumstances in the region and the effects of management actions in the region, then the plan is quickly side-lined from day-to-day operations—as was reported during the assessment of the first plan. This suggests that while the regional plan helps with some management priority setting, in its current form it is insufficiently informative to guide management when circumstances and opportunities change. Further, this suggests that regional plans should focus on the general objectives of principle; they should avoid specific detail, but they will need to be supported by decision support tools and processes that enable the assessment of management options as circumstances change. For this to work, however, current governance and management arrangements would need greater flexibility and commitment to acquiring new skills.

It is evident that the time and effort expended on a paper-based regional NRM plan are mostly of limited value. For those few people strongly involved in the plan development process, there are valuable learning opportunities. For those responsible for writing and submitting the plan, the primary motivation is to meet the criteria set by the State agency. Their predisposition to include new approaches is highly variable. The prospect of trying a different process and acquiring new tools and analyses was viewed as risky and, hence, revision was mostly a refinement of that which already existed. In essence, the development of the regional strategic plan is often viewed as the “end” rather than setting the framework for the compelling connection to or expectation of concerted implementation.

This project extended over a period of more than 4 years, from proposal to post-project demonstration and training. During this time, there were ongoing changes in regional planning and technical staff. Several who were involved in the first planning sessions left the regional organisations while others took different responsibilities. At times during this project, there was uncertainty as to who was representing the regional NRM management. This meant that the rationale and commitment to the original purpose changed as personnel and priorities changed within the NRM Boards. In both NRM regions, the most senior executive was replaced during the project. In essence, the sense of ownership declined as more changes occurred. Hence, the initial commitment to learning changed practice and new tools was overshadowed by the pervasive focus on maintaining a viable structure. In essence, the project had influence in raising awareness of the complexity of futures planning and perhaps because of this, only limited success thus far at introducing a new tool into regional planning. We are yet to have a full assessment of the ongoing value of the LFAT, but almost certainly there will be a need for ongoing interaction and training to build confidence and capability. Evidence from similar decision support systems such as INFFER (Pannell et al. 2012) clearly shows that system support, maintenance and marketing are needed to encourage ongoing use.

The LFAT is publicly available and provides a comprehensive method of capturing the NRM regional information base and clearly demonstrating regional variation. It graphically shows the importance of the locality as potential responses to climate, markets and biodiversity are explored. But we are yet to fully experience how planners and the planning process will use the tool. It is apparent that there are several reasons for this. The issues needing to be addressed are perceived to be complex with no easily implementable actions. There is limited time to explore and learn outside of day-to-day operations. There is resistance to science derived information that is perceived not to be the domain of “on-the-ground” operators and the uncertainty and complexity in any evidence are thought to make planning very difficult in a regulatory environment. It is highly likely that the research team’s involvement with the whole planning process in both NRM regions was too peripheral to substantially change the way the process worked. This situation can be summarised as a failure to develop an in-depth, collaborative relationship. With conditions that limit the capacity to collaborate with any of those involved and with limited time to build trusting relationships, the chances of fulsome “technology transfer” are small.

There appears to be a significant need for the next round of planning to begin by clarifying who the plan is being developed for, how it is expected to be used and how it will be updated to make it a more “living” set of guidelines for actions. In addition, the credibility of the plan will be determined first by the quality of the data and its analysis and second by the strength of ownership by the responsible State Agency, by regional board staff, and by the key community influencers. Apart from a continuing improvement in the scope and detail of the assets of the region, it seems advantageous to simplify the objectives and expectations of outputs.

Much of this project had similar intent and form to several Integrated Landscape Management (ILM) projects in Canada, particularly the Georgia Basin Futures project (Tansey et al. 2002; Robinson et al. 2006; Sheppard et al. 2011) and the Participatory Integrated Assessment of Water Management and Climate Change in the Okanagan Basin, British Columbia (Cohen et al. 2006). All these projects have identified the importance of inclusion, of engagement between land users (implementers), scientists, policy makers, social process specialists, economists and regulators. The importance of transdisciplinary activity is well recognised. The “Framework for participative reflection on the accomplishment of trans-disciplinary research programs” by Roux et al. (2010) provides a basis for assessment that was used by Campbell et al. (2014) to explore how “environmental research could be more influential”. These papers develop a check list of “accountability indicators”, largely from the perspective of the researchers that will be helpful in shaping a successful transdisciplinary research program. Under the heading “users of research” (Campbell et al. 2014), the following indicators are identified:

Capacity for adoption

- Adaptive decision-making and policy revision
- Continuity of personnel
- Co-location of personnel
- Capacity to build upon emerging research

It is evident from the current project that most of these indicators were only partially met. However, changing these to improve the receptiveness, uptake and learning by policy makers and implementers is almost always outside the controlling influence of the research team. A parallel study of the process from the perspective of the “users of research” could provide some much needed insights that would assist future projects of this type.

A comprehensive review of 10 ILM projects in Canada (Bizikova 2009) made the following recommendations for future ILM projects:

- Review current data “to assess their suitability to reflect on changing socio-economic and environmental conditions and their usefulness in envisioning and monitoring future scenarios and policies”
- Establish an independent “board” that represents the various “knowledge communities” to oversee the project
- Effective integration of data and models requires early definition of inputs, outputs and products
- Targeted scientific documents and outputs are important that
- Highlight results and make recommendations
- Provide visual information
- Provide further references
- Involve local and regional networks for effective communication
- Provide learning opportunities

Bizikova (2009) noted that the reviewed projects were able to improve understanding of the connected social, economic and environmental issues in the study areas. However, the projects were all “strongly driven by scientists” and the genuine collaboration with “policy-makers” was limited, and hence by inference, so was the influence on improved sustainability plans and policies. The current project included all the elements identified above but as identified by Roux et al. (2010) and Campbell et al. (2014) the complete, immersed engagement and capacity of end users is as important to the enduring influence of these trans-disciplinary projects as is the quality of the modelling and its outputs. A combination of the “accountability indicators” (Roux et al. 2010) and the recommendations of Bizikova (2009) can provide a comprehensive checklist that can guide ILM projects. However, as Talwar et al. (2011) identified from a comprehensive review of end user involvement in ILM projects “their success will continue to be constrained if not accompanied by changes in the institutional contexts that provide the necessary support and incentives for strong interactivity”.

## Conclusions

It is ironic that the comment from the review of the first strategic plans viz: “both plans were adjudged as not being well informed by the best available science” is repeated in this project. The limited uptake of comprehensive information even with a readily available tool and informed “experts” indicates that the fundamental limitation to acquiring and using the latest science is a chronic problem. Operational staff have too much to do, often have limited expertise and generally receive limited support for doing other than the immediate and necessary. In this setting, ongoing promotion, support and maintenance of new planning tools like LFAT will be needed. It is also likely that the spatial and time complexity of considering possible future land uses in a changing market setting is in itself too complex for ready assimilation into regional planning processes. This project, like many similar ones underestimated the importance of the time and the predisposition capacity needed to form a highly effective collaborative relationship between researchers, end users, policy makers and land users.

Winning over those people who have influence on regional plan development is critical and only comes with mutual agreement on the need to do something different and then only through development of personal trust and openness. Changes in personnel make this need very challenging, particularly if senior leadership and corporate culture are not fully supportive of introducing improved practice. Success in changing planning processes and implementing science-informed natural resource management clearly requires transdisciplinary practices. However, end user involvement, engagement and value expression through envisioning, provision of process-informed analysis, graphical output displays and availability of ongoing support do not necessarily assure changed behaviour in relation to uptake and use of the best available science. Institutional and governance arrangements that are more accepting of the need for adaptive processes and option assessment tools are needed. This applies both to the planning process and to its implementation.
